# Engineering a Carboxyl Methyltransferase for the Formation of a Furan‐Based Bioplastic Precursor

**DOI:** 10.1002/cssc.202300516

**Published:** 2023-06-28

**Authors:** Lucy C. Ward, Ellie Goulding, Daniel J. Rigden, Faye E. Allan, Alessandro Pellis, Harry Hatton, Georg M. Guebitz, Jesus Enrique Salcedo‐Sora, Andrew J. Carnell

**Affiliations:** ^1^ Department of Chemistry University of Liverpool Crown Street Liverpool L69 7ZD United Kingdom; ^2^ Institute of Systems Molecular and Integrative Biology University of Liverpool Crown Street Liverpool L69 7ZB United Kingdom; ^3^ Department of Chemistry and Industrial Chemistry University of Genova via Dodecaneso 31 16146 Genova Italy; ^4^ Institute of Environmental Biotechnology, Department of Agrobiotechnology, IFA-Tulln University of Natural Resources and Life Sciences Vienna Konrad Lorenz Strasse 20 3430 Tulln Austria; ^5^ Austrian Centre of Industrial Biotechnology Konrad Lorenz Strasse 20 3430 Tulln Austria; ^6^ GeneMill, Shared Research Facilities University of Liverpool Liverpool L69 7ZB United Kingdom

**Keywords:** biocatalysis, cascades, carboxylic acids, enzymes, methyltransferase

## Abstract

FtpM from *Aspergillus fumigatus* was the first carboxyl methyltransferase reported to catalyse the dimethylation of dicarboxylic acids. Here the creation of mutant R166M that can catalyse the quantitative conversion of bio‐derived 2,5‐furandicarboxylic acid (FDCA) to its dimethyl ester (FDME), a bioplastics precursor, was reported. Wild type FtpM gave low conversion due to its reduced catalytic efficiency for the second methylation step. An AlphaFold 2 model revealed a highly electropositive active site, due to the presence of 4 arginine residues, postulated to favour the binding of the dicarboxylic acid over the intermediate monoester. The R166M mutation improved both binding and turnover of the monoester to permit near quantitative conversion to the target dimethyl ester product. The mutant also had improved activity for other diacids and a range of monoacids. R166M was incorporated into 2 multienzyme cascades for the synthesis of the bioplastics precursor FDME from bioderived 5‐hydroxymethylfurfural (HMF) as well as from poly(ethylene furanoate) (PEF) plastic, demonstrating the potential to recycle waste plastic.

## Graphical abstract

**Rationally designed carboxyl methyltransferase** FtpM mutant R166M catalyses the dimethylation of 2,5‐furandicarboxylic acid (FDCA) in very high conversion. FtpM R166M was integrated into two different one‐pot enzymatic cascades for conversion of bioderived 5‐hydroxymethylfurfural or poly(ethylene furanoate) plastic to FDCA dimethyl ester (FDME), using oxidases or cutinase respectively.

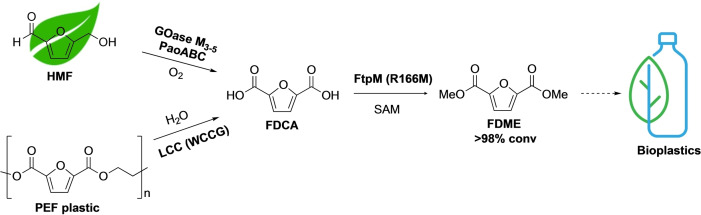

## Introduction

Petrochemical‐derived plastics are omnipresent materials essential to our way of life. However, increasing sustainability pressures require an urgent shift to new alternative materials with a lower carbon footprint, less damaging effect on the environment and that can form part of a circular economy. Bioplastics are plastics that are bio‐based which can also be biodegradable. They are non‐toxic and their production is more sustainable in comparison to traditional plastics.^[1]^ The first synthesised plastic polymers were originally based on natural or renewable materials for example, rubber and celluloid. However, petrochemically derived plastics were developed and became predominant. As a result of environmental pressures and dwindling resources for petrochemical‐derived plastic production, bioplastics are once again at the forefront of addressing the plastics problem.^[2]^ Ideally, new bioplastic materials will be readily recyclable or upcyclable to provide viable end‐of‐life options for spent bioplastic. The global bioplastics market was valued at $9.2 billion in 2020 and is forecast to increase to $47 billion by 2031.^[3]^ Poly(ethylene furanoate) (PEF) has been described as a ‘rising star’ amongst bioplastics. PEF is non‐toxic, recyclable and a bio‐based alternative to poly(ethylene terephthalate) (PET). PEF is made from 2,5‐furandicarboxylic acid (FDCA) **2** which can be formed by the oxidation of bio‐derived 5‐hydroxymethylfurfural (HMF) **1**, itself available from the cellulose component of lignocellulosic biomass.[^4^, ^5^]

Furan derivatives such as FDCA **2** have been referred to as the ‘sleeping giants’ of renewable chemical intermediates as they have high market potential but are generally unexploited.^[6]^ Avantium will complete its new production plant for FDCA in 2024 with an expected capacity of 5.6 kilotonnes/annum.^[7]^ As well as having the advantage of being bio‐derived, PEF also has some improved material properties over PET. In comparison to PET, PEF has a higher gas barrier for O_2_, CO_2_ and H_2_O vapour. It also has improved processability at low temperatures due to its reduced melting temperature (*T*_m_) and can withstand higher temperatures due to a higher glass transition temperature (*T*_g_). This indicates that PEF would be a superior alternative to PET in the packaging materials industry.^[8]^ Avantium have agreed a partnership with Selenis who will produce PEF commercially using the bioderived FDCA.^[9]^ PEF has recently been shown to be succeptible to hydrolysis by cutinases and biobased copolymers that combine FDCA with aliphatic diacids such as poly(butylene adipate‐co‐furandicarboxylate) (PBAF) are compostable.[^10^, ^11^] Thus, furan‐based bioplastics show great promise in terms of thermomechanical properties, excellent barrier properties and biodegradability for the circular economy.^[12]^

**Scheme 1:**
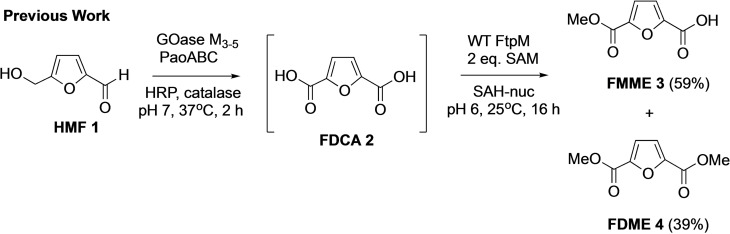
Previously reported one‐pot enzymatic cascade from HMF **1** to FDME **4** using oxidase enzymes^[13]^ and wild type FtpM which gave incomplete conversion to the dimethyl ester FDME **4** required for bioplastic PEF.^[15]^

We have previously developed a one‐pot enzyme cascade process using oxidase enzymes for the conversion of HMF **1** into FDCA **2** in high yield and purity.^[13]^ In order to facilitate product isolation and activate the diacid in situ for further reaction, including polymerisation, we investigated the possibility of using a methyltransferase enzyme for the dimethylation of FDCA **2**. This resulted in the discovery that the carboxyl methyltransferase (CMT) enzyme FtpM, first identified in the natural aromatic fumaric acid amide biosynthetic pathway in *Aspergillus fumigatus*,^[14]^ could methylate a range of mono‐ and dicarboxylic acids including terephthalic acid, 2,5‐pyridine dicarboxylic acid (2,5‐PDCA) and FDCA. This provides an attractive alternative to conventional methyl esterification using classical conditions (MeOH,H^+^, heat) or via the acid chloride/anhydride and allows integration with other enzymatic steps to generate cascade reactions. FtpM showed a preference for diacid substrates over mono carboxylic acids and the first methylation of diacids was much faster than the second methylation. Under optimised conditions we were able to couple FtpM with our previous HMF to FDCA oxidative conversion to demonstrate an extended cascade to obtain 39 % conversion to the diester FDME **4**, starting from HMF **1** (Scheme 1). However, due to the relatively low activity for the second methylation of FDCA **2**, a high FtpM loading was required to achieve sufficient conversion to the dimethyl ester in the final step.^[15]^

**Figure 1:**
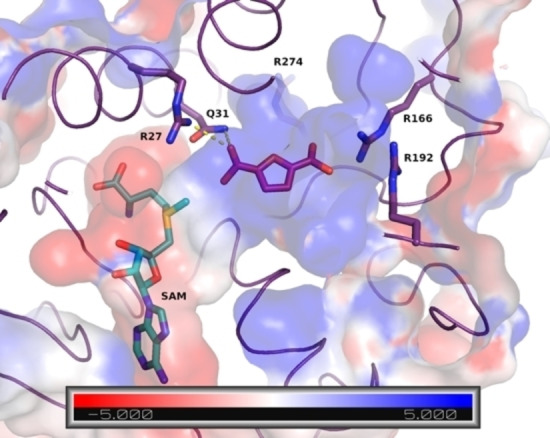
Top‐ranked pose for FDCA **2** (sticks;pink carbon) in the AlphaFold 2 model of the FtpM monomer. The protein is shown as purple ribbon with the surface coloured according to electrostatic calculations carried out with APBS^[16]^ blue positive, red negative; see scale). The unit of the scale is *k*_B_ *T*/*e*_c_ where *k*_B_ is the Boltzmann constant, *T* is the temperature, and *e*_c_ is the charge of the electron. Interactions with Arg27 and Gln31 that position the reactive carboxylic acid group are shown as yellow dashes.

Using an AlphaFold 2 model of FtpM in conjunction with Webina we docked FDCA **2** (Figure 1) and were able to rationalise the kinetic data obtained and the 10‐fold lower specificity constant (*k*_cat_/K_m_) for the second methylation.^[15]^ The carboxylate group to be methylated is correctly oriented by a hydrogen bond to Gln31 and an electrostatic interaction with Arg27. The distance between the carboxylate and the SAM (SAM=*S‐*adenosyl methionine) methyl group (2.9 Å) is sufficient to allow methyl transfer. As a whole, the substrate binding pocket bears strongly positive electrostatic characteristics (R27, R274, R166 and R162) which we postulated would favour binding of the diacid through electrostatic interactions.

We now report that mutagenesis of the active site to reduce the degree of positive charge and increase the hydrophobicity in the region where the initially formed monomethyl ester re‐binds led to improved kinetics for the second methylation of FDCA **2**, while at the same time not significantly affecting the rate of the first methylation. The best mutant was then used to demonstrate a highly efficient cascade for HMF **1** to FDME **4** with >99 % conversion, using a lower concentration of methyltransferase. Mutant R166M also showed improved conversions for selected diacids and monoacid substrates that had previously shown poor conversion. We have also demonstrated an enzymatic cascade for the conversion of PEF plastic to FDME **4** with high efficiency showing the possibility to recycle waste PEF plastic to the starting monomer for re‐polymerisation.

## Results and Discussion

For our selection of mutants, we considered the key residues contributing to the overall positive charge of the substrate binding pocket of FtpM: R27, R274, R166 and R192 (Figure 1). Given the likely importance of its interaction with the reacting carboxylate, R27 was deemed to be a poor choice for mutagenesis. R274 was also not selected as although it contributes to the overall positive charge, it is not in such close proximity to the substrate as R166 and R192, although it could be a potential target in future studies. R166 and R192 were identified as the best target residues for potentially improving monoester binding as they are both located on the side of the unreacted carboxyl group of bound diacid substrate and possess positively charged side chains with favourable electrostatic interaction with the negatively charged carboxylate. R166 was selected as the first residue to be targeted since although R192 may also be a good target, it forms a salt bridge with D188 so mutation may have an adverse impact on enzyme folding. Six single mutants at position R166 were selected to test the effects of charge and hydrophobicity, these were R166F, I, M, Q, H and K.

**Figure 2:**
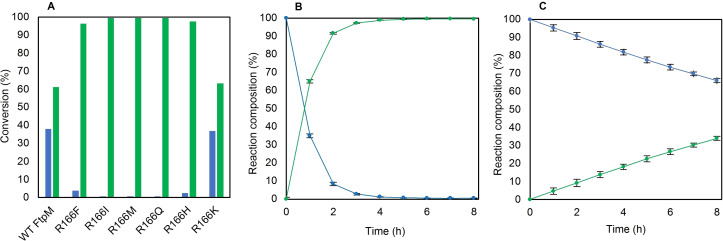
A) Results of assaying FtpM R166X mutants with FMME **3** using 1 mM substrate, 500 μM enzyme, 2 mM S‐adenosyl methionine (SAM) and 4 μM SAH‐nuc (SAH‐nuc=*S*‐adenosylhomocysteine nucleosidase) in 50 mM 2‐(*N*‐morpholino)ethanesulfonic acid (MES) buffer pH 6.0. Reactions were incubated at 25 °C for 16 h. Products detected via reverse phase HPLC (RP‐HPLC). B) 8 h time course for FtpM R166M and WT FtpM with FMME (C), conditions as for (A). Products detected via RP‐HPLC. Blue: FMME **3**; green: FDME **4**.

Site directed mutagenesis of WT FtpM was performed using the original wild type construct as the template. The mutants were expressed using the optimised procedure developed for WT FtpM.^[15]^ It appeared that mutagenesis at this residue did not have a significant negative effect on protein yield in most cases (Table S1). The 6 mutants were initially tested with the furan monomethyl ester FMME **3** under the reaction conditions established for WT FtpM. We were pleased to observe that 5 mutants had significantly improved activity with FMME **3**, with conversions to 2,5‐furandicarboxylic acid dimethyl ester, FDME **4** of over 97 % (Figure 2A). On kinetic analysis (Table 1), K_m_ values for hydrophobic substitutions (R166I and R166M) showed improved binding for FMME **3**. A 9‐fold increase in *k*_cat_ for R166M coupled with the most significant improvement in binding lead to the greatest improvement in catalytic efficiency (*k*_cat_/K_m_). A time course reaction with FMME **3** using R166M showed a marked improvement over the wild type (Figure 2B vs Figure 2C). The significant improvement observed for R166M for methylation of FMME **3** supports our hypothesis that replacing the charged arginine with the less polar methionine side chain facilitates improved binding and turnover of the monomethylated compound where the methyl ester group can be accommodated in a less polar environment. An alternative explanation is that with the WT enzyme, the monoester **3** binds in a non‐productive mode with the carboxylate interacting with R166, possibly competing with FDCA **2**. Mutating R166 to a less polar residue allows monoester **3** to bind in a productive mode for methyl transfer from SAM. When we examined FDCA **2** as the substrate with each mutant, only three mutants (I, M and Q) gave an overall higher conversion for dimethylation of FDCA **2** to FDME **4** (Figure S19). This was disappointing and suggested that the beneficial mutations for the second methylation may be deleterious for the binding of the diacid for the first methylation. R166K was the only mutant to have a significant decrease in conversion to FDME **4** (22 %) in comparison to WT FtpM. Interestingly, in all cases there was significant (>5 %) FDCA **2** remaining in comparison to WT FtpM (0.5 %). Notably, with R166M, which achieved the highest conversion to FDME (65 %), 25 % FDCA **2** remained present after 16 h.

**Table 1:** Kinetic parameters of WT FtpM and FtpM R166X mutants with FMME **3** and FDCA **2**. Michael‐Menten curves including standard error bars can be found in the Supporting Information (Figures S2–S13).

Enzyme	Substrate	K_m_ [mM]	*k*_cat_ [min^−1^]	*k*_cat_/K_m_ [mM^−1^ min^−1^]
WT FtpM	**3**	1.40	0.005	0.004
FtpM R166F	**3**	0.97	0.015	0.015
FtpM R166I	**3**	0.84	0.016	0.019
FtpM R166M	**3**	0.64	0.045	0.070
FtpM R166Q	**3**	0.77	0.021	0.027
FtpM R166H	**3**	1.31	0.019	0.015
FtpM R166K	**3**	1.41	0.006	0.004
WT FtpM	**2**	0.52	0.022	0.042
FtpM R166F	**2**	0.85	0.027	0.032
FtpM R166I	**2**	0.47	0.024	0.051
FtpM R166M	**2**	0.66	0.024	0.036
FtpM R166Q	**2**	0.67	0.027	0.040
FtpM R166H	**2**	0.73	0.020	0.027
FtpM R166K	**2**	0.55	0.017	0.031

When starting with FDCA **2**, kinetic analysis (Table 1) shows that most mutants (except R166I) had a lower binding affinity (higher K_m_) for FDCA compared to WT FtpM. R166K had the most similar K_m_ to the WT enzyme (0.55 and 0.52 mM respectively) which is likely a result of the lysine residue maintaining a positive charge and the slight increase in K_m_ may be because lysine is less basic than arginine. However, the overall catalytic efficiency (*k*_cat_/K_m_) is lower than WT FtpM as a result of a lower *k*_cat_ value. The increase in K_m_ for most of the other mutants supports the idea that a positive charge at position R166 is beneficial to diacid binding. This includes the R166H mutant which has an increased K_m_ value (0.73 mM) either because the shorter side chain prevents interaction with the unreacted carboxylate group of the diacid, because at pH 6 only half of the histidine residues are protonated and/or because the pKa of the histidine is reduced due to the highly positive environment. There were slight variations between the *k*_cat_ values for the 6 mutants with FDCA, but no significant differences compared with WT FtpM.

The R166X mutants with the highest catalytic efficiencies with FDCA **2** were R166I, R166M and R166Q. However, these kinetic parameters did not correlate with the conversions obtained by HPLC when using FDCA **2** as a substrate (Figure S19). R166M had a catalytic efficiency of 0.036 mM min^−1^ with FDCA as a substrate which was not significantly different to WT FtpM (0.042 mM min^−1^) but when tested with 1 mM FDCA there was 25 % substrate remaining after 16 h, which would suggest that the activity with FDCA **2** was severely affected. To further understand these results, a time course study of FtpM R166M versus WT FtpM with 1 mM FDCA **2** was performed (Figure S20). With R166M this showed that after 4 h, the reaction did not proceed further, explaining the 25 % remaining diacid after 16 h reaction.

**Figure 3:**
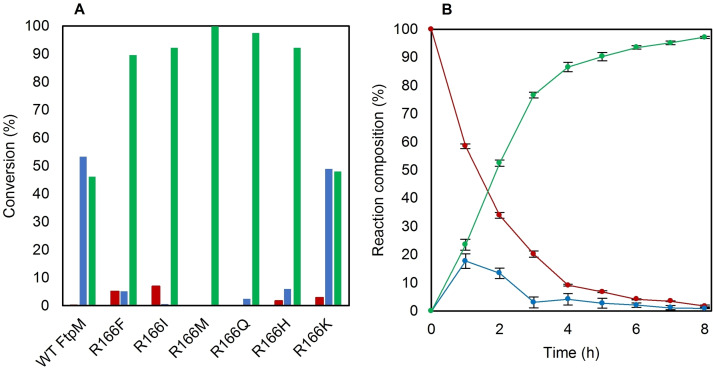
A) Results of assaying FtpM R166X mutants with FDCA **2** using 1 mM substrate, 500 μM enzyme, 4 mM SAM and 8 μM SAH‐nuc in 50 mM MES pH 6.0. Reactions were incubated at 25 °C for 16 h. Products detected via RP‐HPLC. B) 8 h time course for FtpM R166M with FDCA, conditions as for (A). Red: FDCA; blue: FMME; green: FDME.

Given the proven increased efficiency for FMME **3** to FDME **4** conversion with this mutant, we hypothesised that the reaction when starting with FDCA **2** may be limited by supply of the cofactor SAM. Although it had been shown previously with WT FtpM that doubling the concentration of SAM from 2 mM to 4 mM did not improve the conversion of FDCA,^[15]^ with R166M and the other mutants (except R166K) we found that use of 4 mM (2 equiv.) gave much higher conversions (Figure 3A) to FDME **4**, with R166M achieving near quantitative (>99 %) conversion after 16 h.

**Scheme 2:**
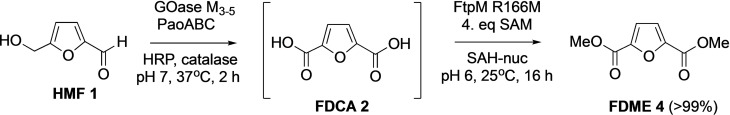
HMF **1** to FDME **4** cascade. Reaction consisted of 1 mM HMF, 1.85 μM GOase M_3‐5_, 3.7 μM PaoABC, 0.17 mg mL^−1^ catalase and 0.11 mg mL^−1^ HRP in 100 mM KPi pH 7.0 at 37 °C for 2 h. The pH was dropped to 6.0 and 200 μM FtpM R166M, 4 mM SAM and 8 μM SAH‐nuc were added with incubation at 25 °C for 16 h. Products detected via RP‐HPLC.

The time course reaction when using 4 mM SAM showed that the higher rate of FDME **4** formation was maintained up to around 80 % conversion (Figure 3B). It is possible that the mutant has improved kinetic parameters with SAM in comparison to the wild type enzyme. However, the results strongly indicate that the increased rate of the second methylation results in all available SAM being used, as the reaction with R166M stops after 4 h whereas it is still proceeding after 8 h with the WT FtpM (Figure S20A) when using limited (2 equiv.) SAM cofactor. With an improved mutant in hand for efficient conversion of FDCA **2** to FDME **4**, we applied this to our previously reported cascade for a one pot enzymatic conversion of bioderived HMF **1** to bioplastics precursor FDME **4**. Using a reduced loading of FtpM R166M (200 μM), we were able to achieve >99 % conversion of HMF **1** to FDME **4** (Scheme 2) compared with the previous maximum yield of 39 % (Scheme 1).

In addition to FDCA **2** and FMME **3** we also tested selected other mono and dicarboxylic acids previously shown to undergo methylation with the WT enzyme^[15]^ to probe the ability of the R166M mutant to deliver improved conversion. Terephthalic acid **5**, pyridine 2,5‐dicarboxylic acid (2,5‐PDCA) **8** and isophthalic acid **12** all gave significantly higher conversion to the dimethyl esters compared to the WT (Table 2). 2,5‐PDCA **8** can be made from lignin biomass using engineered whole cells of *Rhodococcus jostii* RHA1 and simple esters have been used as bioplastic precursors.[^17^, ^18^, ^19^] The apparent preference for methylation of **8** at the 2‐position by the mutant to give 2‐methyl ester **9** compared to the WT might also be explained by increased methylation of 5‐methyl ester **10** by the mutant to give diester **11**. With the selection of monoacids tested we found that conversions were also significantly improved (**15**, **17**, **23**, **25**) or the high conversions previously obtained with the WT were maintained (**19** and **21**). With 3‐OH and 3‐MeO benzoic acids **19** and **21** we were able to reduce the amount of enzyme to 200 μM and still obtain >99 % conversion to the methyl ester.

**Table 2:** Conversions to ester products catalysed by FtpM R166M and WT FtpM. 1 mM substrate, 500 μM enzyme, 2 mM SAM and 4 μM SAH‐nuc in 50 mM MES pH 6.0. Reactions were incubated at 25 °C for 16 h. Products detected via RP‐HPLC.

Substrate	FtpM R166M		WT FtpM	
	Monomethyl ester [%]	Dimethyl ester [%]	Monomethyl ester [%]	Dimethyl ester [%]
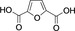	**3** (3^[b]^)	**4** (>99^[a][b]^)	**3** (48)	**4** (44)
**2**				
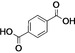	**6** (3^[b]^)	**7** (96^[b]^)	**6** (58)	**7** (36)
**5**				
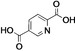	2‐Me ester **9** (55^[b]^) 5‐Me ester **10** (1^[b]^)	**11** (41^[b]^)	2‐Me ester **9** (17) 5‐Me ester **10** (65)	**11** (17)
**8**				
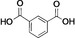	**13** (4^[b]^)	**14** (95^[b]^)	**13** (46)	**14** (52)
**12**				
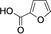	**16** (72)		**16** (23)	
**15**				
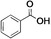	**18** (76)		**18** (46)	
**17**				
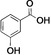	**20** (99^[a]^)		**20** (89)	
**19**				
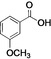	**22** (99^[a]^)		**22** (99)	
**21**				
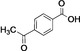	**24** (43)		**24** (22)	
**23**				
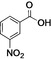	**26** (40)		**26** (16)	
**25**				

[a] Enzyme concentration lowered to 200 μM. [b] SAM and SAH‐nuc concentration doubled. Compound numbers are in bold, conversions are in brackets.

**Scheme 3:**
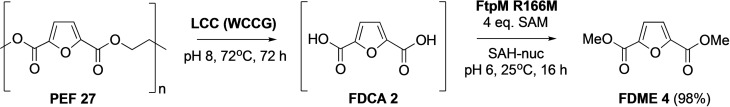
PEF **27** to FDME **4** cascade. Reaction consisted of 5 mg PEF, 10 μM LCC(WCCG) in Tris‐HCl buffer pH 8.0 at 72 °C for 72 h. The PEF reaction was then diluted to contain a final concentration of 1 mM FDCA, 200 μM FtpM R166M, 4 mM SAM, 8 μM SAH‐nuc in MES buffer pH 6.0 at 25 °C for 16 h. Products detected by RP‐HPLC.

We also demonstrated that FDCA **2** could be sourced from biobased plastic PEF **27** (which could be an effective recycling strategy for blends and multilayers containing PEF), by incorporating the improved methylation of FDCA **2** by FtpM R166M into a two‐step enzymatic cascade from PEF **27** to FDME **4** (Scheme 3). Cutinase from leaf‐branch compost (LCC) has emerged as a promising candidate for PET degradation due to its ability to hydrolyse PET directly to terephthalic acid and ethylene glycol.^[20]^ An engineered LCC mutant (WCCG) has recently been described to have increased depolymerization activity in comparison to WT LCC, so we investigated this variant for the hydrolysis of the PET analogue PEF **27**.[^20^, ^21^] PEF degradation has previously been reported with cutinases Thc_cut1 and HiC giving 12.8 mM FDCA from PEF films over 96 h.^[10]^ Using LCC (WCCG), we achieved 11 mM FDCA **2** from 5 mg of finely milled low molecular weight PEF **27** at pH 8.0 after 72 h (Figure S21). Due to the requirement of 4 equivalents of SAM cofactor for the dimethylation of 11 mM FDCA **2** by FtpM R166M, the reaction was diluted to contain a final concentration of 1 mM FDCA **2** in the FtpM R166M methylation step to demonstrate the cascade. Using the optimised reaction conditions for methylation of FDCA **2** with FtpM R166M, we were able to achieve 98 % conversion to FDME from the 1 mM FDCA **2** formed by PEF degradation in a one‐pot, two‐step cascade. This cascade has allowed us to not only describe a promising candidate for PEF hydrolysis with LCC (WCCG), but also demonstrates the valorisation of PEF degradation product FDCA **2**, through transformation to bioplastics precursor FDME **4**. Further processing would allow FDME **4** to either reform PEF or be converted to other bioplastics such as PBAF. In the light of these results we also envisage that terephthalate and isophthalate produced by polymer recycling methods (chemical and enzymatic) could be recycled by enzymatic methylation.

## Conclusion

The ability to enzymatically methylate carboxylic acids and diacids in aqueous buffer in high conversion allows this key step for activation to be integrated into enzyme cascades for multistep conversions. In our original characterisation of the carboxyl methyltransferase FtpM we noted the slow rate of the second methylation for the key substrate 2,5‐furandicarboxylic acid, FDCA **2**, to give the dimethyl ester FDME **4** (FDME=2,5‐furandicarboxylic acid dimethyl ester), a key intermediate for the synthesis of bioplastic poly(ethylene furanoate), PEF **27**. A high enzyme loading was also required due to its overall low activity. We previously demonstrated an enzyme cascade from bioderived 5‐hydroxymethylfurfural, HMF **1** to FDME **4** in modest 39 % overall conversion, which was a result of the slow rate for the second methylation catalysed by WT FtpM.^[15]^ AlphaFold2 was used to generate a highly accurate predicted structure of FtpM which identified that the substrate binding pocket showed strong electropositive characteristics. This suggested that while the active site may be good for binding diacids, with the arginine 166 side chain forming a favourable electrostatic interaction with the non‐reacting acid group, following the initial methylation and re‐binding of the monomethyl ester, the arginine side chain interaction may be less favourable, slowing the second methylation. R166 was subjected to mutagenesis with the aim to improve kinetics for the second methylation, in the hope that the first methylation could still occur at a sufficient rate. Of the six R166 mutants tested, we found that R166M gave the best (9‐fold) increase in *k*_cat_ with a significant improvement in binding, resulting in the largest increase in catalytic efficiency for the second methylation. Although there was a slight lowering of catalytic efficiency for the diacid FDCA **2**, use of the R166M mutant for the double methylation still permitted higher overall conversion to dimethylated product even at a lower (200 μM) FtpM concentration. Three other R166 mutants (I, Q and H) also gave >90 % conversion to FDME **4**. Incorporation of R166M into the one‐pot cascade showed >99 % conversion for HMF **1** to FDME **4**. We also demonstrated an additional cascade in which FDCA **2** was generated from PEF plastic. Additionally, we have shown that the R166M mutant shows improved conversion of other diacids and monoacids compared to the WT enzyme and that a lower enzyme loading can be used.

Current efforts are focussed on improving the overall catalytic efficiency of the enzyme for future biotechnology applications within whole cells. While there are several recently published systems for in vitro SAM cofactor recycling[^22^, ^23^] it is likely that larger scale application of carboxyl methyltransferase enzymes would involve use of recombinant strains in which SAM recycling can be upregulated. This will facilitate in situ capture of enzymatically synthesised carboxylic acids, negating the need for use of stoichiometric base as is usual for bioprocesses producing acids. The enzymatic production of methyl esters facilitates isolation of the more hydrophobic product esters by partitioning into organic solvent overlays.^[21]^ Uptake of doubly charged diacids has been demonstrated using whole cells of an engineered *E. coli* strain for conversion of terephthalic acid to vanillin. The uncharged vanillin product could be isolated by in situ‐product removal (ISPR) using an oleyl alcohol overlay, minimizing any product toxicity.^[24]^ Thus, a similar approach could be envisioned for whole cell bioconversions using FtpM. Biomethylation of carboxylic acids also allows for further coupled reactions as part of synbio pathways (e. g., amidation/transesterification using promiscuous acyl transferases).^[25]^

## Experimental Section

### Plasmids and strains

Codon optimised genes of WT FtpM (Q4WZ45), SAH‐nucleosidase (P0AF12), and LCC (PETH_UNKP) with the WCCG mutation (F243W/D238C/S283C/Y127G)^[26]^ were synthesised by GeneArt and subcloned into a pET‐based golden gate vector with a C‐terminally coded histidine tag. LCC (WCCG) plasmid also contained a PelB tag. PaoABC subunit genes in the PMN100 plasmid derived from pTrcHisA (Invitrogen) was provided by S. Leimkuhler, University of Potsdam.^[27]^ FtpM R166X mutants were engineered from the WT FtpM plasmid and mutagenesis was performed by Azenta Life Sciences, Japan.

Catalase (EC 1.11.1.6). from bovine liver, lyophilized powder with a specific activity of 2000–5000 U mg^−1^ protein was purchased from Sigma Aldrich, Gillingham, UK. Peroxidase from horseradish (EC 1.11.1.7), type VI, lyophilized powder with a specific activity of >250 U mg^−1^ protein was purchased from Sigma Aldrich, Gillingham, UK. Freeze dried cell lysate expressing GOase M_3‐5_ was purchased from Prozomix Limited, Haltwhistle, UK.

### Protein expression and purification

Production of SAH‐nucleosidase, WT FtpM, PaoABC and GOase M_3‐5_ (purification only) were as described previously.^[15]^

FtpM R166X mutants were transformed into *E. coli* SoluBL21 (DE3) cells (Genlantis). A single colony was added to 10 mL LB medium supplemented with 100 μg mL^−1^ ampicillin and incubated at 37 °C, 120 rpm for 16 h. This was used as a starter culture to inoculate the main culture (1 L LB medium supplemented with 100 μg mL^−1^ ampicillin) which was incubated at 37 °C, 180 rpm until the culture had reached an OD_600_ of 0.6–0.8. Gene expression was then induced by the addition of 1 mM IPTG and the temperature was reduced to 16 °C and incubated for a further ≈16 h with aeration.

LCC (WCCG) was transformed into *E. coli* BL21 (DE3) cells (Invitrogen). A single colony was added to 10 mL LB medium supplemented with 100 μg mL^−1^ ampicillin and incubated at 37 °C, 120 rpm for 16 h. This was used as a starter culture to inoculate the main culture (1 L terrific broth autoinduction medium supplemented with 100 μg mL^−1^ ampicillin) which was incubated at 22 °C, 180 rpm for 24 h.

For both preparations, the cells were harvested (4000 xg, 10 min, 4 °C) and the cell pellet was resuspended in resuspension buffer (50 mM sodium phosphate pH 7.4, 300 mM NaCl) and lysed using sonication. Cell debris was removed by centrifugation (15000 xg, 1 h, 4 °C) and the supernatant was filtered using a 0.45 μM syringe filter prior to loading onto a HisTrap Fast Flow affinity column (GE Healthcare) equilibrated with resuspension buffer. The column was washed with resuspension buffer containing 50 mM imidazole and protein elution was performed using resuspension buffer containing 500 mM imidazole.

Purified protein was buffer exchanged into 50 mM MES pH 6.0 (FtpM R166X mutants) or equilibration buffer (LCC (WCCG)) using a PD‐10 Sephadex column (GE Healthcare) and protein concentration was increased using a centrifugal filter unit centrifuged at 4000 xg for 10 min.

### WT FtpM and FtpM R166X mutant reaction conditions

Unless otherwise stated, FtpM R166X mutant reactions were set up to contain a final concentration of 8 μM SAH‐nucleosidase, 4 mM SAM (NEB), 500 μM FtpM R166X and 1 mM substrate in 50 mM MES buffer pH 6.0. Reactions were incubated for 16 h at 25 °C with shaking at 180 rpm. Control reactions were also set up parallel to assay reactions but excluding the addition of FtpM mutant. An equivalent volume of 10 % triflouroacetic acid (TFA) was added after reaction incubation and centrifuged at 13000 rpm, 3 min in a table‐ top centrifuge to precipitate out and remove any protein prior to subsequent analysis.

### HMF to FDME cascade conditions

HMF to FDME cascade conditions using WT FtpM were as described previously.^[15]^

HMF to FDME cascade conditions using FtpM R166M: reactions were set up to contain a final concentration of 1 mM HMF, 1.85 μM GOase M3‐5, 3.7 μM PaoABC, 0.17 mg mL^−1^ catalase from bovine liver and 0.11 mg mL^−1^ horseradish peroxidase in 100 mM KPi buffer pH 7.0. Reactions were incubated for 2 h at 37 °C with shaking at 250 rpm. After 2 h, the pH was dropped to 6.0 using HCl and 8 μM *S*‐adenosylhomocysteine (SAH) nucleosidase (SAH‐nuc), 4 mM *S‐*adenosyl methionine (SAM) (NEB), 200 μM FtpM R166M were added, and reactions were incubated for 16 h at 25 °C with shaking at 180 rpm. An equivalent volume of 10 % triflouroacetic acid (TFA) was added after reaction incubation and centrifuged at 13000 rpm, 3 min in a table‐ top centrifuge to precipitate out and remove any protein prior to subsequent analysis.

### PEF degradation conditions

PEF reactions were set up to contain 5 mg low molecular weight PEF and 10 μM LCC(WCCG) in 100 mM Tris‐HCl buffer pH 8.0 or 9.0 (500 μL reaction volume). Reactions were incubated for 72 h at 72 °C with shaking at 100 rpm. A 50 μL sample was taken after 72 h and the enzyme heat inactivated at 120 °C for 3 min and centrifuged at 13000 rpm, 3 min in a table‐top centrifuge. 2 % (TFA) was added to reaction samples before being diluted x8 with MeOH prior to analysis by RP‐HPLC to determine the FDCA concentration using a calibration curve (Figure S22). This was then multiplied by the dilution factor to determine the final concentration of FDCA following the biotransformation.

### PEF to FDME cascade conditions

PEF reactions were set up to contain 5 mg low molecular weight PEF and 10 μM LCC(WCCG) in 100 mM Tris‐HCl buffer pH 8.0 (500 μL reaction volume). Reactions were incubated for 72 h at 72 °C with shaking at 100 rpm. A 50 μL sample was taken after 72 h, the enzyme heat inactivated at 120 °C for 3 min and centrifuged at 13000 rpm, 3 min in a table‐top centrifuge. A sample was taken and analysed as in 1.4.3 to determine the final FDCA concentration. A sample was then taken from the reaction mixture to give a final concentration of 1 mM FDCA as a substrate for the methylation step. 200 μM FtpM R166M, 4 mM SAM (NEB) and 8 μM SAH‐nucleosidase were also added and the volume made up to 100 μL with 50 mM MES buffer pH 6.0. Reactions were incubated for 16 h at 25 °C with shaking at 180 rpm. An equivalent volume of 10 % triflouroacetic acid (TFA) was added after reaction incubation and centrifuged at 13000 rpm, 3 min in a table top centrifuge to precipitate out and remove any protein prior to subsequent analysis.

### RP‐HPLC conditions

All Reverse‐phase HPLC was performed using an Agilent 1260 Infinity system equipped with a 4.6×150 mM ZORBAX Eclipse XDB‐C18 5 μM column (Agilent). 5 μL of sample was injected and run on a gradient of 0.1 % TFA in 5 : 95 methanol/water to 0.1 % TFA in 90 : 10 methanol/water at a flow rate of 0.6 mL min^−1^ for 30 min at 35 °C. To fully quantify the final conversion of substrate to product, HPLC peak areas were adjusted using a 1 : 1 standard of substrate/product(s) or a calibration curve of substrate/product concentration was created. RP‐HPLC retention times can be found in Table S2.

### Kinetics

Kinetic parameters were established using the previously reported method for WT FtpM kinetic measurements.^[15]^ Michaelis–Menten curves are shown in Figures S2–S13.

### Computational methods

Protein models of the FtpM monomer and dimer structures were modelled using AlphaFold 2^[28]^ as descried previously.^[15]^

### Synthesis of PEF

The synthesis of PEF was performed as previously reported by Pellis et al.^[29]^ two‐stage melt‐polycondensation method was used. Briefly, a two necked round bottom flask equipped with a distillation bridge was used as reaction vessel. Three N_2_/vacuum cycles were performed to remove all moisture from the reaction environment before starting the reaction. 2,5‐furandicarboxylic acid dimethyl ester (FDME) and 1,2‐ethylene glycol (EG) were added in a 1 : 3 (FDME/EG) molar ratio. 450 ppm (calculated in respect to the mass of the diester) of tetrabutyl titanate were added. The mixture was gradually heated to 160 °C while the whole apparatus was kept under an N_2_ atmosphere. The temperature was kept for 2 h. The temperature was then increased to 170 °C and kept constant for 2 h. Afterwards, the temperature was increased to 190 °C for another 2 h. In the second step of polycondensation, a vacuum of 0.1 mbar was applied. The temperature was increased to 220 °C and kept for 2 h, followed by a last temperature increase to 235 °C which was kept for 4 h. To recover the reaction product, the apparatus was cooled down to room temperature and the product was recovered from the reaction vessel as a white solid. The material was ground in a mortar before further analysis.

## Conflict of interest

The authors declare no conflict of interest.

1

## Supporting information

As a service to our authors and readers, this journal provides supporting information supplied by the authors. Such materials are peer reviewed and may be re‐organized for online delivery, but are not copy‐edited or typeset. Technical support issues arising from supporting information (other than missing files) should be addressed to the authors.

Supporting Information

## Data Availability

The data that support the findings of this study are available in the supplementary material of this article.
