# Supplementary material for: Engineering a Carboxyl Methyltransferase for the Formation of a Furan‐Based Bioplastic Precursor
Source: ChemSusChem. 2023 Jun 28;16(16):e202300516. doi: 10.1002/cssc.202300516 (PMC10946451; doi:10.1002/cssc.202300516)
Supplement: Supplementary file 1 — Supporting Information [file CSSC-16-0-s001.pdf]

ChemSusChem

Supporting Information

Engineering a Carboxyl Methyltransferase for the Formation of a Furan-Based Bioplastic Precursor

Lucy C. Ward, Ellie Goulding, Daniel J. Rigden, Faye E. Allan, Alessandro Pellis, Harry Hatton, Georg M. Guebitz, Jesus Enrique Salcedo-Sora, and Andrew J. Carnell*© 2023 The Authors. ChemSusChem published by Wiley-VCH GmbH. This is an open access article under the terms of the Creative Commons Attribution License, which permits use, distribution and reproduction in any medium, provided the original work is properly cited.

Table of Contents

1. Experimental Procedures 3

1.1 Protein expression 3

1.2 RP-HPLC retention times 4

1.3 Kinetics 6

1.4 Synthesis of PET 10

1.4.1 NMR analysis 10

1.4.2 TGA analysis 11

1.4.3 DSC analysis 12

1.4.4 GPC Analysis 12

2. Results and Discussion 13

2.1 FtpM R166X mutant conversions with FDCA (2 mM SAM) 13

2.2 WT FtpM and FtpM R166M time courses with FDCA (2 mM SAM) 13

2.3 LCC (WCCG) catalysed PEF degradation tests 14

References 14

Author Contributions 14

1. Experimental Procedures

1.1 Protein expression

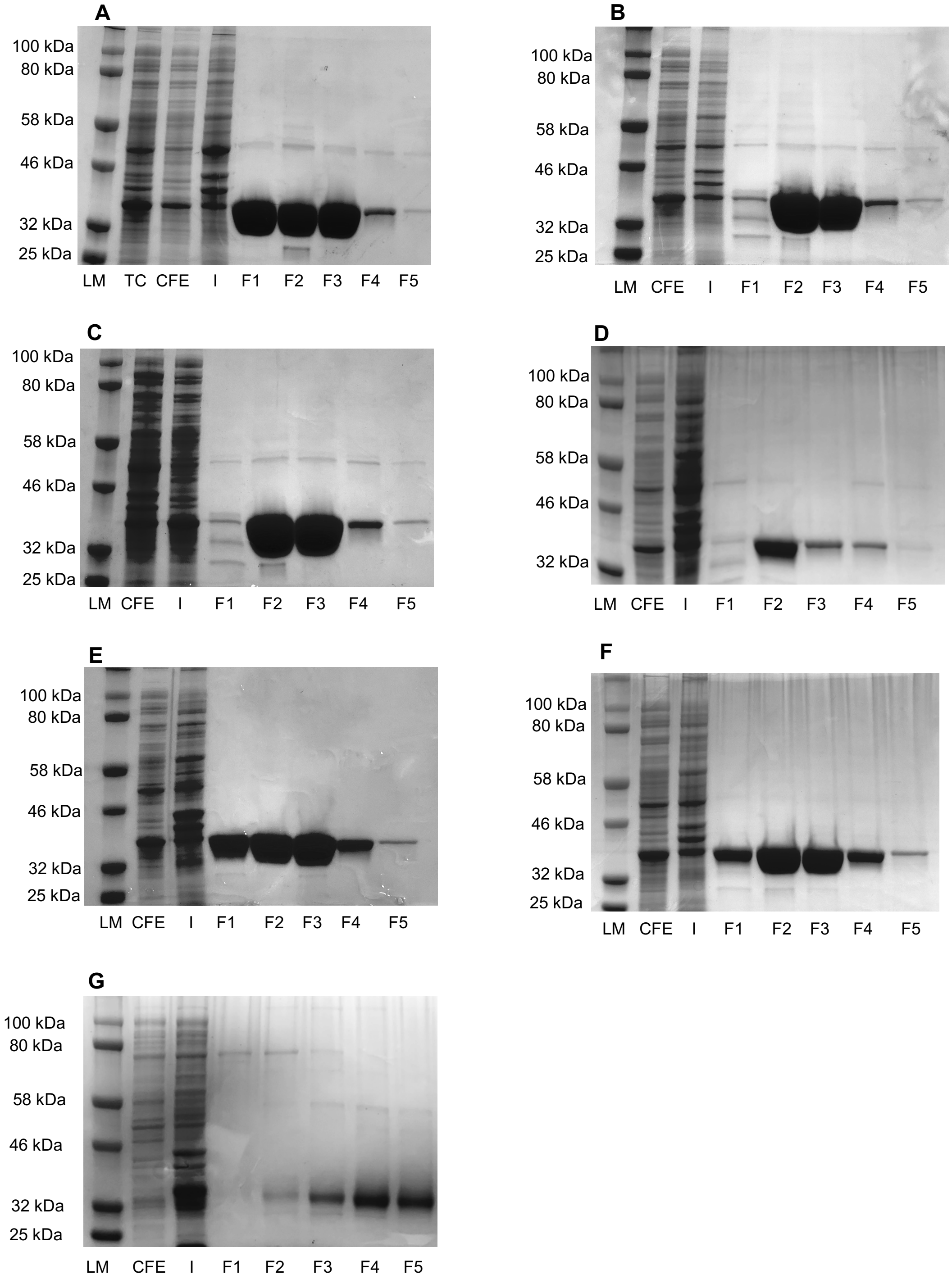

Figure S1 10% SDS-PAGE separation of protein purification fractions. LM: lane marker, TC: total cellular content, CFE: cell free extract, I: insoluble fraction, F: eluted protein fractions. (A) FtpM R166F, (B) FtpM R166I (C) FtpM R166H (D) FtpM R166K (F) FtpM R166M (F) FtpM R166Q, (G) LCC (WCCG).

Table S1 Pure protein yields of WT FtpM and FtpM R166X mutants from 1 L LB culture.

**Enzyme**	**Protein yield (mg)**
WT FtpM	>60[1]
FtpM R166F	64
FtpM R166H	76
FtpM R166I	82
FtpM R166K	50
FtpM R166M	73
FtpM R166Q	66

1.2 RP-HPLC retention times

Table S2 RP-HPLC retention times. RP-HPLC conditions described in manuscript.

Entry	Time (min)
**1**	9.6
**2**	16.3
**3**	19.4
**4**	21.6
**5**	20.2
**6**	23.5
**7**	25.8
**8**	12.8
**9**	18.4
**10**	19.1
**11**	21.0

**12**	20.8
**13**	23.2
**14**	25.3
**15**	15.1
**16**	19.4
**17**	21.9
**18**	24.7
**19**	18.8
**20**	22.0
**21**	23.6
**22**	25.4
**23**	21.2
**24**	23.7
**25**	22.4
**26**	24.5

Compounds **1** and **10** were purchased from Fluorochem. Compound **2** was purchased from Alfa Aesar. Compound **3** was synthesised in-house as described previously.[1] Compounds **4** and **9** were purchased from Apollo Scientific. Compound **8** was kindly provided by Dr Thomas Farmer, University of York. Compounds **11**-**24** were purchased from Sigma-Aldrich.

1.3 Kinetics

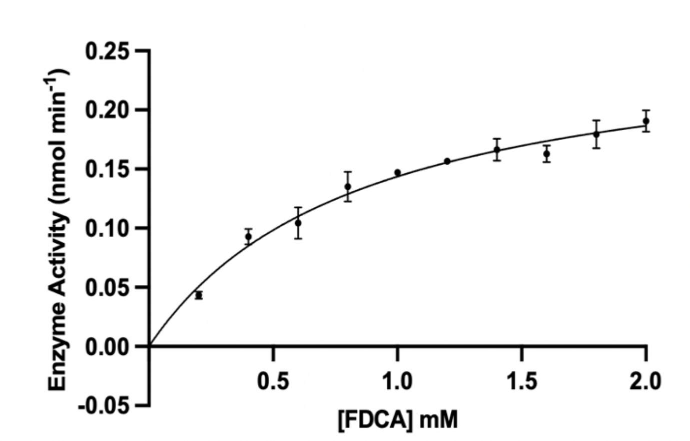

Figure S2 Michaelis-Menten curve used to determine kinetic parameters of the methylation of FDCA **2** catalysed by FtpM R166F.

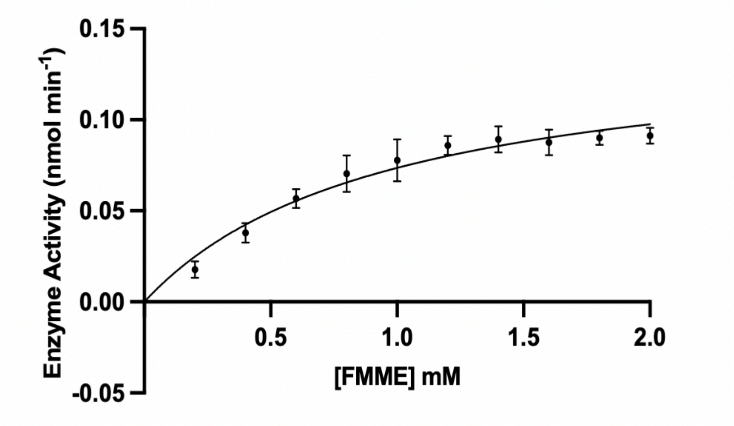

Figure S3 Michaelis-Menten curve used to determine kinetic parameters of the methylation of FMME **3** catalysed by FtpM R166F.

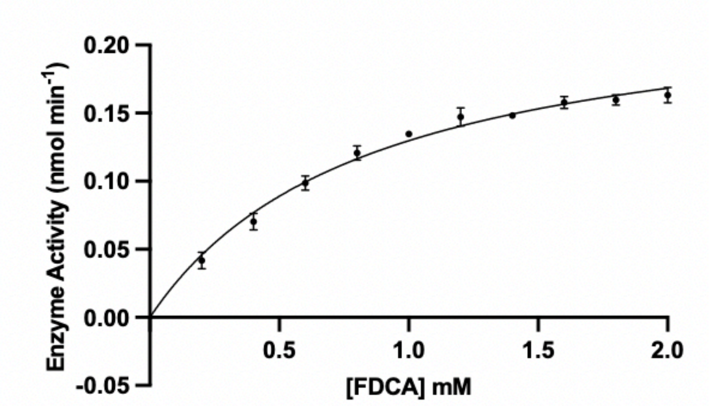

Figure S4 Michaelis-Menten curve used to determine kinetic parameters of the methylation of FDCA **2** catalysed by FtpM R166I.

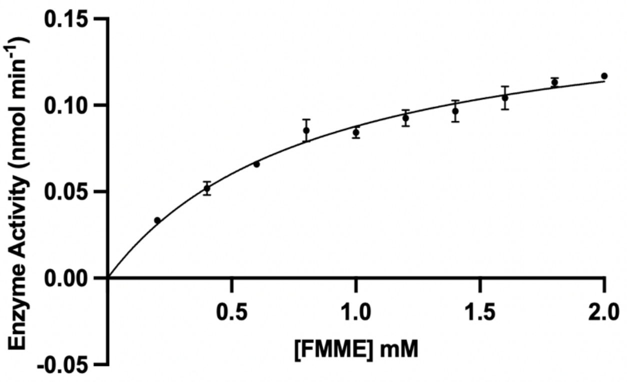

Figure S5 Michaelis-Menten curve used to determine kinetic parameters of the methylation of FMME **3** catalysed by FtpM R166I.

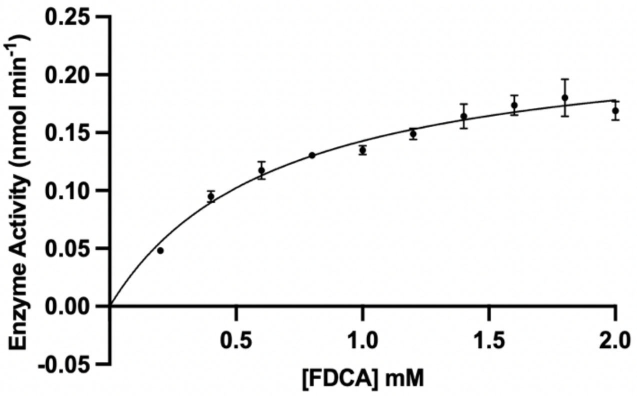

Figure S6 Michaelis-Menten curve used to determine kinetic parameters of the methylation of FDCA **2** catalysed by FtpM R166M.

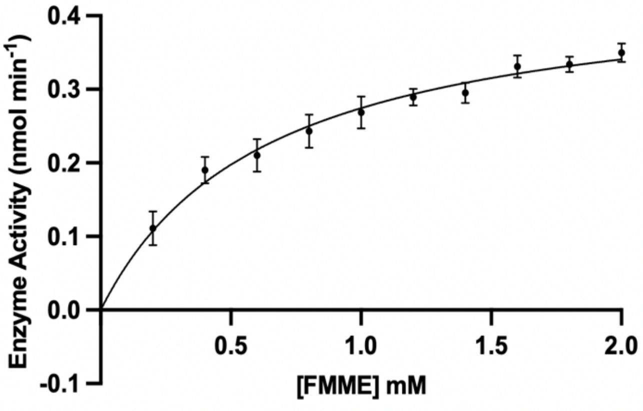

Figure S7 Michaelis-Menten curve used to determine kinetic parameters of the methylation of FMME **3** catalysed by FtpM R166M.

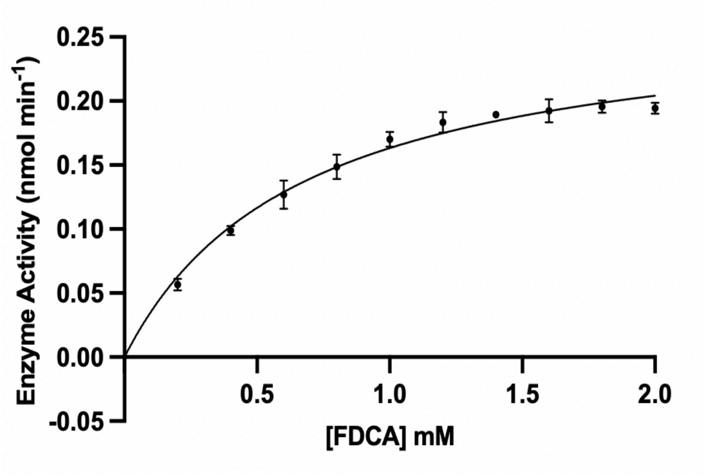

Figure S8 Michaelis-Menten curve used to determine kinetic parameters of the methylation of FDCA **2** catalysed by FtpM R166Q.

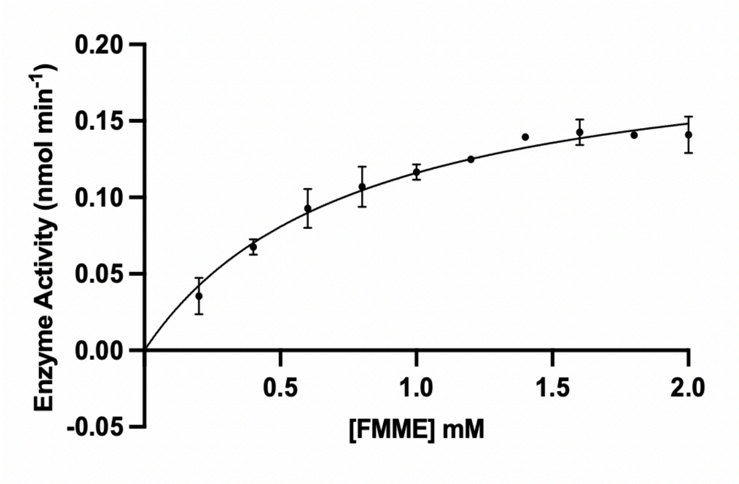

Figure S9 Michaelis-Menten curve used to determine kinetic parameters of the methylation of FMME **3** catalysed by FtpM R166Q.

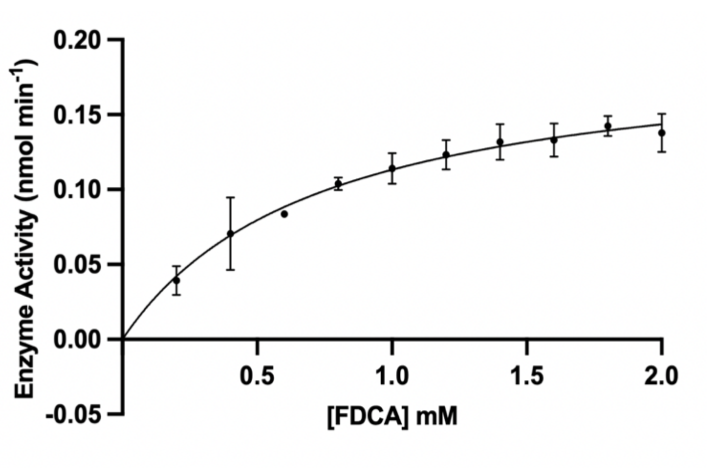

Figure S10 Michaelis-Menten curve used to determine kinetic parameters of the methylation of FDCA **2** catalysed by FtpM R166H.

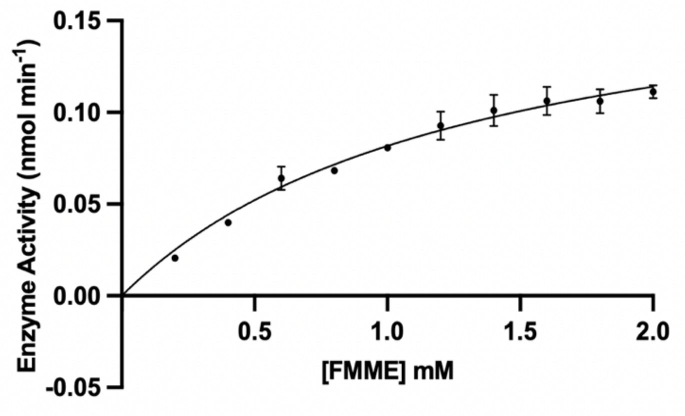

Figure S11 Michaelis-Menten curve used to determine kinetic parameters of the methylation of FMME **3** catalysed by FtpM R166H.

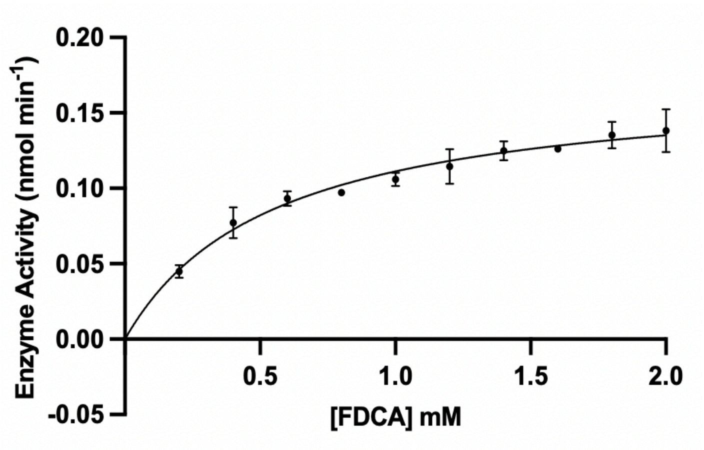

Figure S12 Michaelis-Menten curve used to determine kinetic parameters of the methylation of FDCA **2** catalysed by FtpM R166K.

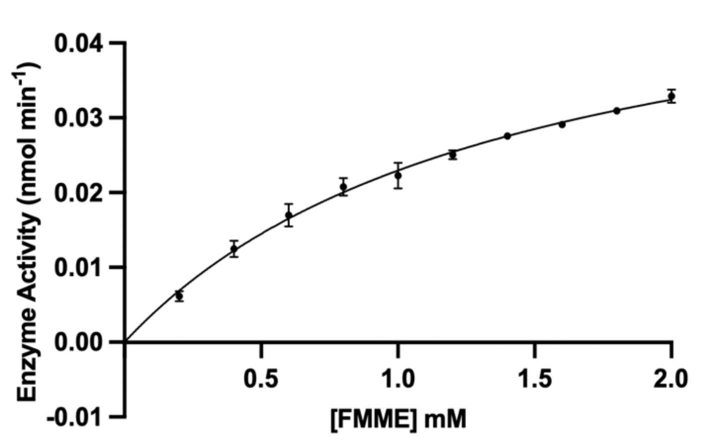

Figure S13 Michaelis-Menten curve used to determine kinetic parameters of the methylation of FMME **3** catalysed by FtpM R166K.

1.4 Synthesis of PET

1.4.1 NMR analysis

^{1}H, ^{13}C and HSQC NMR spectroscopy characterizations were performed using a JEOL ECZ400R/S3 and a $CDCl_3$/TFA-d mixture as the solvent.

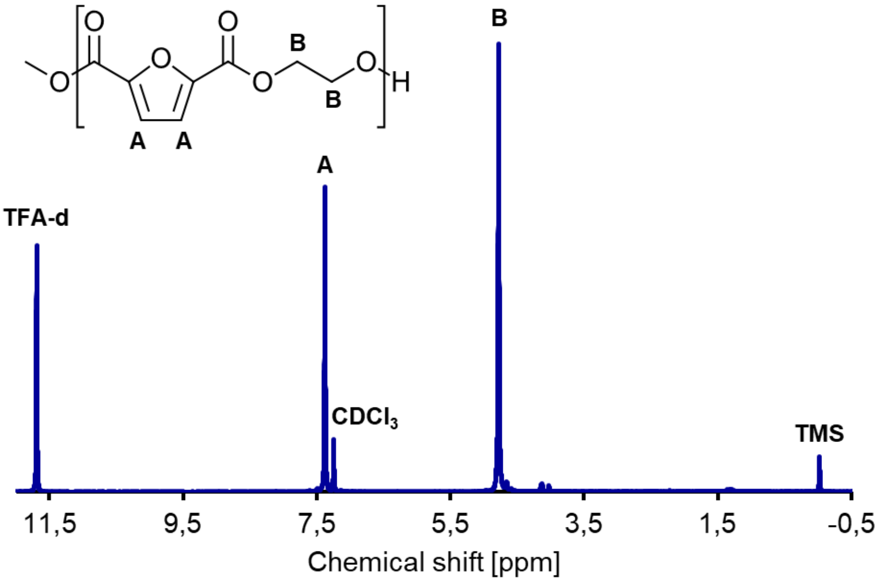

Figure S14 ^{1}H-NMR analysis of the synthesized PEF.

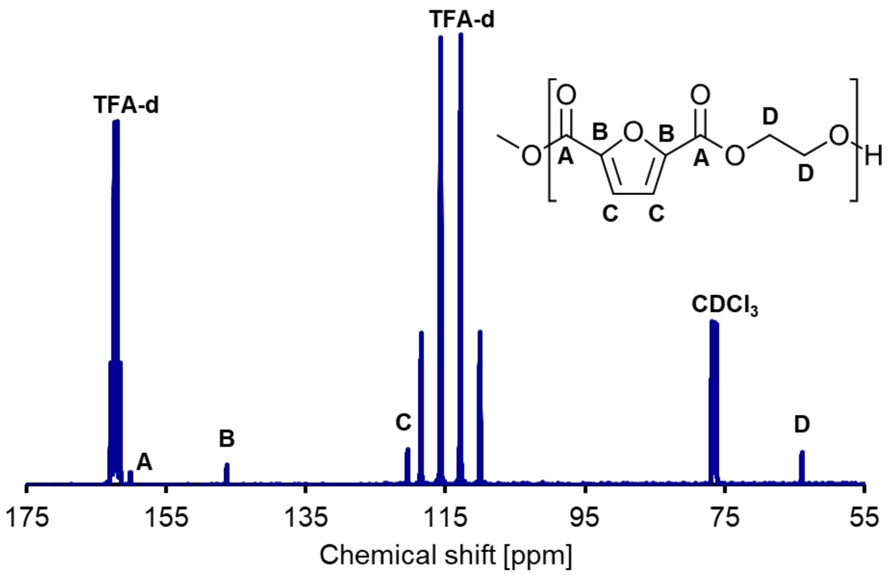

Figure S15 ^{13}C-NMR analysis of the synthesized PEF.

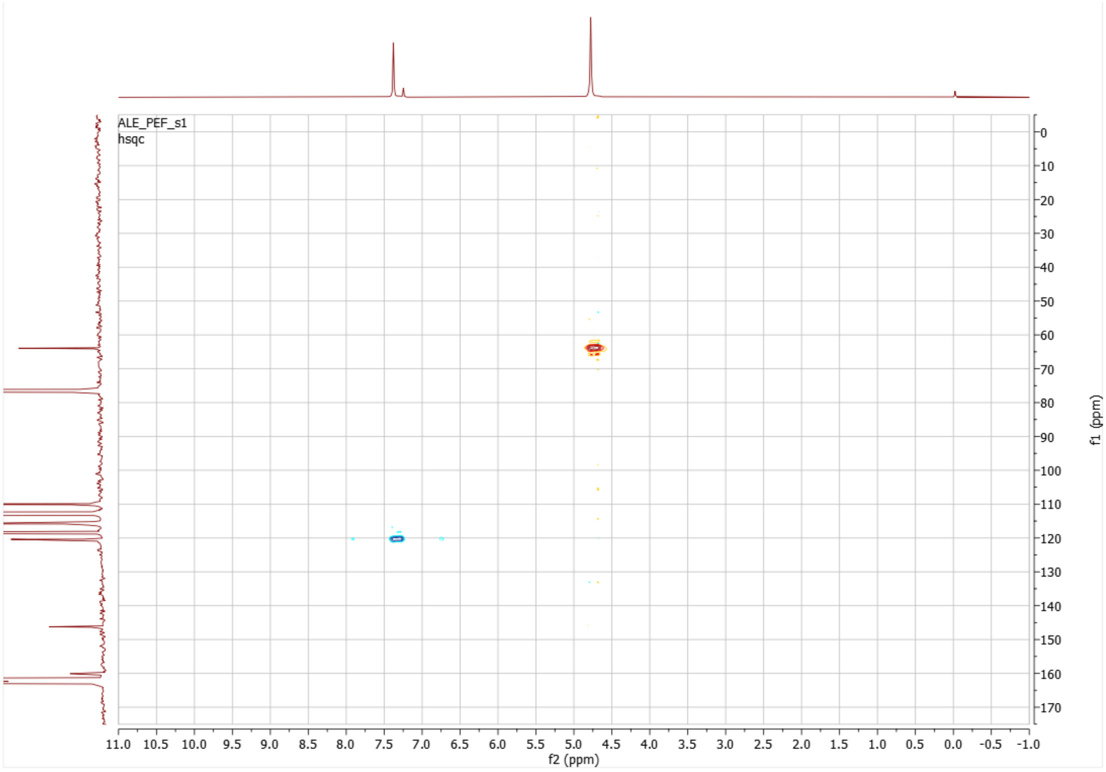

Figure S16 HSQC analysis of the synthesized PEF.

1.4.2 TGA analysis

Thermogravimetric analysis (TGA) was performed using a Mettler Toledo TGA/DSC1 STARe System® using a 25-800°C temperature range with a heating rate of 10°C min^{-1}, under an 80 mL min^{-1} N_2 flow.

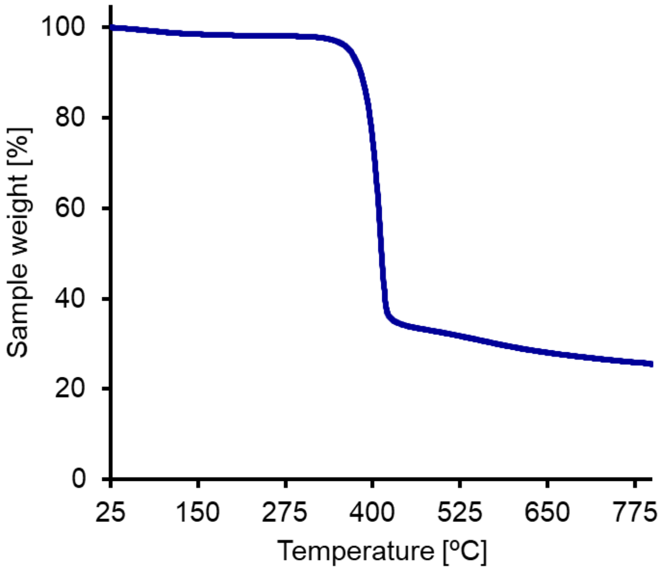

Figure S17 Thermogravimetric analysis of the synthesized PEF.

1.4.3 DSC analysis

Differential Scanning Calorimeter (DSC) thermograms were measured using a Mettler Toledo "DSC1 STARe System®" in the −40-240°C temperature range using a heating/cooling rate of 10°C min^{-1}, with a N_2 flow of 20 mL min^{-1}.

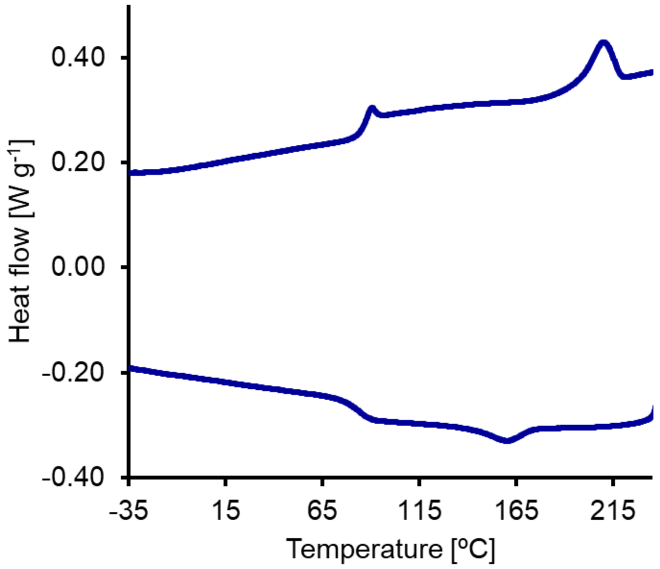

Figure S18 Differential scanning calorimetry analysis of the synthesized PEF (1st cooling and 2nd heating cycle).

1.4.4 GPC Analysis

Gel permeation chromatography (GPC) was run on a different PEF sample which was prepared using the same method described above to give the following molecular weight data: Mn= 7400 g mol^{-1}, Mw= 18900 g mol^{-1}, Mw/Mn= 2.54. GPC was performed on a system equipped with a Waters 1515 Isocratic HPLC pump, a Waters 2414 refractive index detector (35°C), a Waters 2707 auto sampler, and a PSS PFG guard column followed by two PFG-linear-XL (7 µm, 8 × 300 mm) columns in series at 40°C. Hexafluoroisoproanol (HFIP) with potassium trifluoroacetate (3 g L^{-1}) was used as the eluent at a flow rate of 0.8 mL min^{-1}. The molecular weights were calculated against poly(methyl methacrylate) standards.

2. Results and Discussion

2.1 FtpM R166X mutant conversions with FDCA (2 mM SAM)

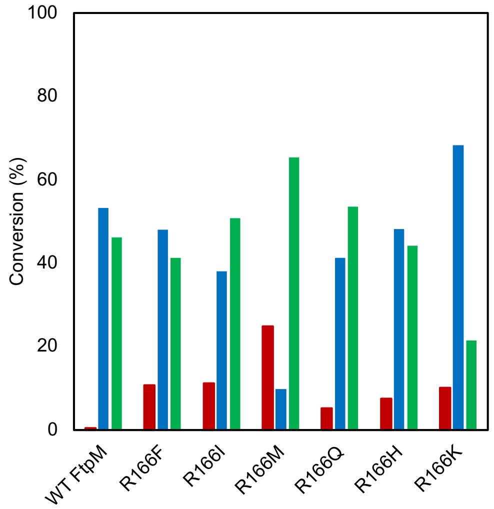

Figure S19 Results of assaying FtpM R166X mutants with FDCA **2** using 1 mM substrate, 500 µM enzyme, 2 mM SAM and 4 µM SAH-nuc in 50 mM MES pH 6.0. Reactions were incubated at 25°C for 16 h. Products detected via RP-HPLC. Red: FDCA; blue: FMME; green: FDME.

2.2 WT FtpM and FtpM R166M time courses with FDCA (2 mM SAM)

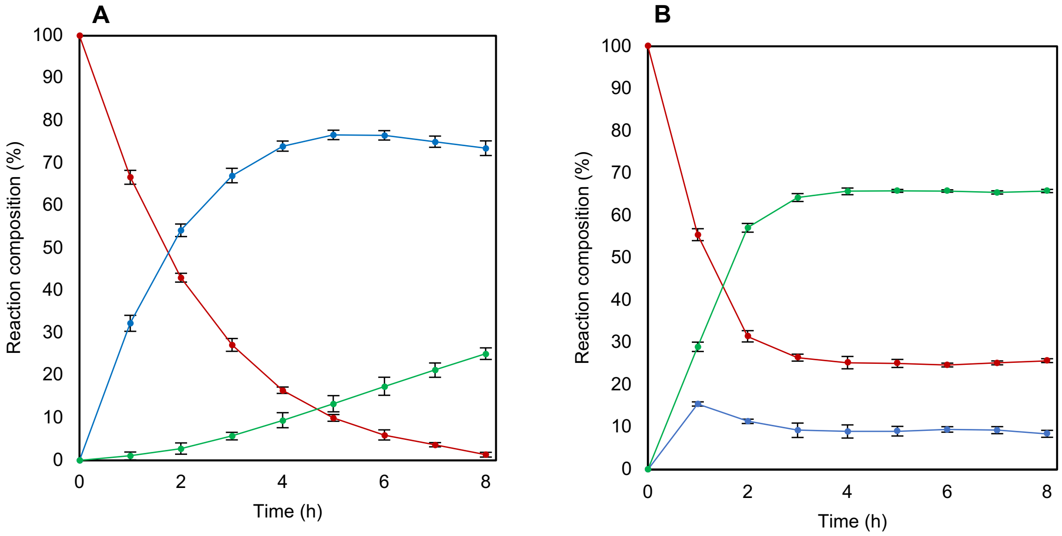

Figure S20 8 h time courses for WT FtpM (**A**) and FtpM R166M (**B**) with FDCA using 1 mM substrate, 500 µM enzyme, 2 mM SAM and 4 µM SAH-nuc in 50 mM MES pH 6.0. Reactions were incubated at 25°C for 16 h. Products detected via RP-HPLC. Red: FDCA; blue: FMME; green: FDME.

2.3 LCC (WCCG) catalysed PEF degradation tests

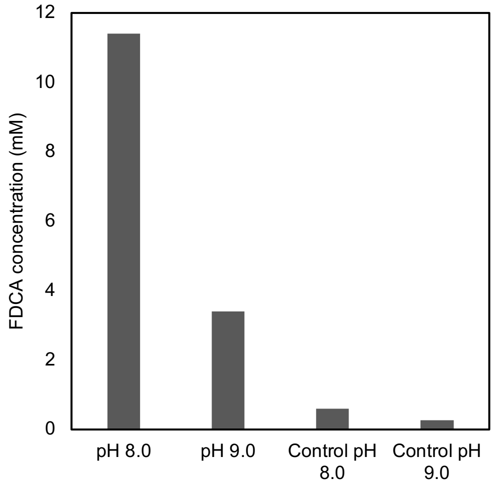

Figure S21 PEF hydrolysis to FDCA at pH 8.0 and pH 9.0. Reactions consisted of 5 mg low molecular weight PEF and 10 µM LCC (WCCG), incubated at 72°C for 72 h. Products detected via RP-HPLC. Controls excluded addition of LCC (WCCG). Final FDCA concentration was determined as stated in **1.4.3**.

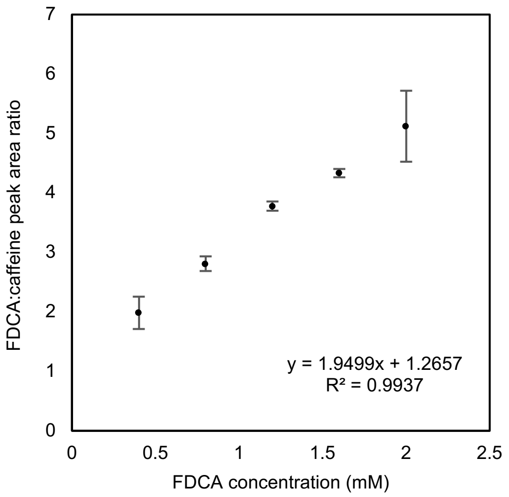

Figure S22 FDCA calibration curve. A range of standards consisting of different concentrations of FDCA were analysed via RP-HPLC with 1 mM caffeine as an internal standard. The average FDCA: caffeine peak area ratio was plotted against FDCA concentration to generate the curve.

References

[1] L. C. Ward, H. V. McCue, D. J. Rigden, N. M. Kershaw, C. Ashbrook, H. Hatton, E. Goulding, J. R. Johnson, A. J. Carnell, *Angew. Chemie - Int. Ed.* **2022**, *61*, e202117324.

Author Contributions

AJC devised the project and acquired funding. AJC and JESS supervised the project. DJR modelled the WT FtpM structure and aided mutant selection. LCW expressed the R166X mutants, characterized their activity, performed kinetic studies and performed the HMF to FDCA cascade. FEA completed the substrate scope analysis with FtpM R166M. EG performed the PEF degradation studies and achieved the PEF cascade to FDCA. AP carried out the synthesis and characterisation of the PEF under the supervision of GMG. EG and HH synthesised the FMME standard. LCW, AJC and EG wrote the manuscript and generated the figures.
